# *Pichia pastoris* is a Suitable Host for the Heterologous Expression of Predicted Class I and Class II Hydrophobins for Discovery, Study, and Application in Biotechnology

**DOI:** 10.3390/microorganisms6010003

**Published:** 2018-01-05

**Authors:** Julie-Anne Gandier, Emma R. Master

**Affiliations:** Department of Chemical Engineering and Applied Chemistry, University of Toronto, Toronto, ON MSE 3E5, Canada; emma.master@utoronto.ca

**Keywords:** hydrophobin, *Pichia pastoris*, immunoassay, protein expression, fermentation, *Cordyceps militaris*

## Abstract

The heterologous expression of proteins is often a crucial first step in not only investigating their function, but also in their industrial application. The functional assembly and aggregation of hydrophobins offers intriguing biotechnological applications from surface modification to drug delivery, yet make developing systems for their heterologous expression challenging. In this article, we describe the development of *Pichia pastoris* KM71H strains capable of solubly producing the full set of predicted *Cordyceps militaris* hydrophobins CMil1 (Class IA), CMil2 (Class II), and CMil3 (IM) at mg/L yields with the use of 6His-tags not only for purification but for their detection. This result further demonstrates the feasibility of using *P. pastoris* as a host organism for the production of hydrophobins from all Ascomycota Class I subdivisions (a classification our previous work defined) as well as Class II. We highlight the specific challenges related to the production of hydrophobins, notably the challenge in detecting the protein that will be described, in particular during the screening of transformants. Together with the literature, our results continue to show that *P. pastoris* is a suitable host for the soluble heterologous expression of hydrophobins with a wide range of properties.

## 1. Introduction

Filamentous fungi thrive in a wide range of environments throughout their life cycle. This ability can be attributed not only to the plasticity of their metabolism, but also to the often-overlooked non-catalytic proteins that are secreted and implicated in their adaptations to the surrounding environment. As the primary decomposers of all terrestrial ecosystems [1], these organisms produce enzymes that have been extensively studied for their ability to convert lignocellulosic biomass to soluble sugars for biofuels and biochemicals applications. The importance of non-catalytic, or weakly hydrolytic, accessory proteins in this process has also been considered. For instance, swollenins [2,3,4], cerato-platanins [5], and loosenins [6] have been implicated in cellulose disruption, which can increase accessibility to enzymes and improve conversion.

There is increasing interest in screening non-catalytic fungal proteins for a broader range of applications. For instance, exclusively found in filamentous fungi of the Ascomycota and Basidiomycota phyla, hydrophobin proteins self-assemble to tailor interfaces to fungal life. These small 7–20 kDa proteins associate to coat and reverse the wettability of air-exposed fungal surfaces, such as spores [7,8] and fruiting bodies [9], serving roles in protection and adhesion [10]. They assemble at the air–liquid interface to reduce the surface tensions of liquid media allowing for the emergence of aerial structures [11]. In pathogenic fungi, they have been shown to act as virulence factors assembling on the host’s surface to recruit enzymes, facilitate fungal adhesion, and/or generate the hydrophobic surface-signal necessary for the development of the infection structures [12]. While a wide range of roles has been proposed, the underlying feature shared amongst these proteins is interfacial assembly.

These biological functions have inspired a number of biotechnological applications, which are currently at various stages of development (reviewed in [13,14,15]). Their uses have been explored for the dispersal of hydrophobic solids, liquids, and air in applications such as food foam stabilization and drug delivery [16,17,18,19]. Certain hydrophobins have been used in the laboratory to coat surfaces for the selective immobilization of cells [20] and molecules, such as protein [21]. Conversely, others have been applied as antifouling films [22]. These seemingly contradictory abilities highlight the palette of surface properties that can be produced depending on the choice of hydrophobin and solution conditions for a given application.

Most broadly, this palette can be divided into two classes (I and II) with distinct biophysical properties and sequence features. Class I hydophobins self-assemble at interfaces to form highly stable films that can only be disassociated by strong acids, such as trifluoroacetic acid or formic acid. These films take on a regular “rodlet” morphology with an amyloid-like structure. Class II assemblies are comparatively less stable and can be disassociated by detergent-alcohol mixtures [23].

**Figure 1:**
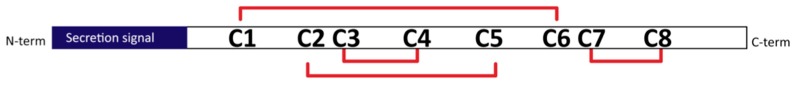
The primary sequences of hydrophobins are characterized by a pattern of eight cysteines (C) numbered one through eight that form four disulfide bridges in a conserved pattern (red brackets). C2 and C3 along with C6 and C7 are positioned together in the sequence.

**Figure 2:**
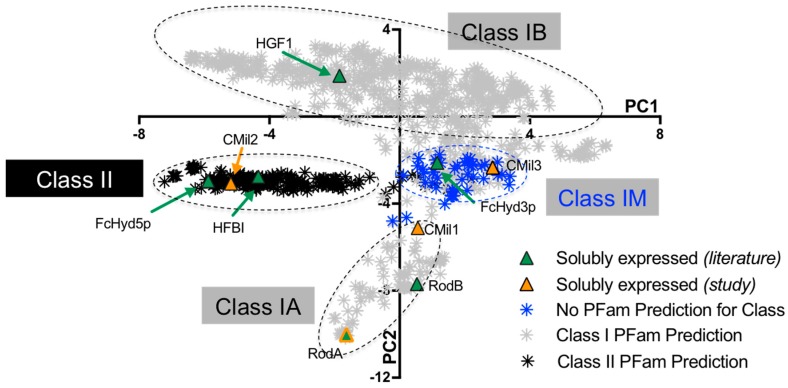
Clustering of hydrophobins previously expressed (green) and expressed in the current study (orange) by the host *Pichia pastoris* in the context of the sequence alignment principal component analysis (SA-PCA) as reported in [25]: an analysis of the confirmed and predicted hydrophobin protein sequences from the genomes of over 200 fungal species. The SA-PCA serves as a visual representation of the protein sequence database from which the proteins studied herein were selected. The dashed circles draw attention to the different regions validated by multiple protein sequence alignments (see ref. [25]) as well as PFam predictions (colour of the marker). Neither the position of these circles nor their size were calculated by means of the analysis of the PCA results. The SA-PCA is a useful visual representation, as the sequences that cluster together along a principal component tend to share similarities in their predicted primary protein sequences, the degree of similarity for a given group increasing with distance from the origin of the graph. The clustering along the first principal component (PC1) highlights the most significantly different clusters in the context of the total data. While the differences described in the second principal component (PC2) are less significant in the context of the total data, it achieves the subdivision of Class I hydrophobins: two high-similarity groups, A and B, as well as group M whose subdivisions within Class I are yet to be understood. PFam predictions of the sequences are indicated by the colour of the markers (Class I in grey, Class II in black, no prediction in blue). Sequences located in the Class II and Class IA regions are of Ascomycota origin and those in the Class IB region are of Basidiomycota origin. The sequences in the IM region are a mixture of both phyla.

Considered together, the primary sequences belonging to the hydrophobin family share little similarity except for a conserved motif of eight cysteines with characteristic disulfide bridging (Figure 1) [24]. Similarity emerges when examining hydrophobin classes individually. While Class II sequences are relatively well-conserved, it is in fact Class I hydrophobins that share little to no similarity despite the shared solution and interfacial properties described above [25]. Our recent work explains the high diversity of Class I sequences [25]. To better contextualize the primary structures studied herein, we provide a visual representation of this analysis (i.e., a sequence alignment principal component analysis) as it was conducted in [25] in Figure 2. Briefly, we constructed a database of confirmed and predicted hydrophobins from the genomes of over 200 fungal species. The principal component analysis (PCA) of the protein sequence alignment matrix of the database combined with PFam domain assignations revealed two high-identity Class I subdivisions (verified by protein sequence alignments) [25]: Class IA, containing sequences of Ascomycota origin, and Class IB, containing those of Basidiomycota origin. The Class I sequences that remain are a mixture of Ascomycota and Basidiomycota sequences that share far lower similarity and lie at the interface of these two groups. We refer to these as IM sequences (of mixed phylum), and their subdivisions within Class I are yet to be understood. These groups can be visualized in the plot of the first and second principal components of the sequence analysis in Figure 2 as it was conducted in [25]. We refer to this graph as the sequence alignment principal component analysis (SA-PCA).

The principal distinguishing feature of the Class I subdivisions is the spacing of the cysteines in their conserved motif as described in Figure 1; more specifically, the spacing between the third and fourth cysteines as well as the fourth and fifth cysteines is strikingly different. In Class IA, these regions are on average, respectively, 40 and 22 residues in length, while in Class IB they are substantially shorter, measuring on average 32 and 13 residues, respectively [25]. It is important to note that there have not been specific solution properties experimentally associated to these sequence features. Class IA is defined purely on sequence similarity. In the case of Class IB, however, it was experimentally determined that the monomers of these proteins likely share a three-dimensional structure: the structure of the *Schizophyllum commune* hydrophobin was resolved and is believed to be representative of group IB sequences.

The aim of the herein study was to establish an expression and detection strategy for the recombinant production of predicted and confirmed hydrophobin genes from across the spectrum described in the SA-PCA. Ultimately, this strategy would be applied to produce proteins whose function and biophysical characteristics could be studied to not only inform the role of hydrophobins in the biology of filamentous fungi, but also to provide a “palette” of these interface-active proteins for biotechnological applications [26]. Ideally, this would be conducted using strategies that are readily scalable.

A significant challenge to the industrial application of hydrophobins is their production at a corresponding scale. Currently, two modified hydrophobins are available, which are produced at pilot-scale (order of the kg) by BASF (Baden Aniline and Soda Factory) and marketed as a foam stabilizer [27]. Referred to as H*ProteinA and H*ProteinB, they consist of the Class I hydrophobin DewA (*Aspergillus nidulans*) with a C-terminal 6His-tag, and each have a different solubilisation protein tag at the N-terminus. They are produced in *Escherichia coli* and purified from the inclusion bodies in a system extensively optimized specifically for this construct.

In contrast, if hydrophobins are produced solubly, it circumvents the need to purify the protein from inclusion bodies and avoids the use of strong acids, such as trifluoroacetic acid, to solubilize, for instance, the aggregates of Class I proteins. The Ascomycota yeast *Pichia pastoris* has been extensively used as a host for the heterologous expression of fungal proteins from the lab bench to industrial scale. Furthermore, it has been demonstrated to solubly express hydrophobins (Table 1). Its use is currently approved both for pharmaceutical and food applications and so can readily be scaled for industrial application in a wide range of sectors. Furthermore, *P. pastoris* is characterized by its ability to perform diverse post-translational modifications, including glycosylation and folding, via the chaperone characteristics of fungi-derived proteins. Its high levels of productivity in an almost protein-free medium combined with the ability to engineer secreted proteins allow for the purification of a given protein from the growth medium without the need to harvest the cells themselves, offering the possibility of semi-batch and continuous processes [28]. As such, if the pipeline were to be constructed using the *P. pastoris* expression system, it would be readily scalable.

## 2. Materials and Methods

### 2.1. Protein Sequence Analyses

Protein sequences examined in the context of this study were selected from the database constructed in [25], which consists of 1046 predicted and confirmed hydrophobin protein sequences from the genomes of 215 fungal species. Briefly, the UniProt, JGI, and NCBI databases were mined for the canonical eight cysteine pattern residue (Figure 1). The resulting sequences were submitted to signal [29] using default parameters to determine the predicted cleavage site of the secretion signal and to PFam [30] for domain assignation (i.e., Class I, Class II, or No predicted class).

Class I subdivisions (IA, IB, or IM) were assigned to hydrophobins based on previous analyses of the above-described protein database [25]. Briefly, a multiple protein sequence alignment was constructed from the protein sequences. Principal component analysis (PCA) was then applied to the protein sequence alignment matrix as a means of visually representing the similarity of sequences. The PCA was conducted using Jalview (version 2.8.2) using the BLOSUM62 score matrix option [31]. Superimposing PFam predictions on PC2 versus PC1 (as in Figure 2) then conducting protein sequence alignments of the different regions allowed for the identification of regions A and B. This work is shown in [25]. Region M sequences are at the interface of group A and B and represent sequences with highly variable distances between the cysteines of the characteristic eight-cysteine pattern. Their subdivision in Class I is yet to be understood.

The sequence alignments presented in the figures herein were conducted using the software Geneious version 8.1.5 [32]. The ClustalW alignment algorithm with the BLOSUM cost matrix was used either with or without free end gaps (option reported with result).

Grand average hydropathy (GRAVY) scores were calculated as described in [33], and β-aggregator scores were obtained using the TANGO algorithm [34].

### 2.2. Strain Construction and Expression Level Screening

Predicted and confirmed hydrophobin protein coding sequences were codon optimized for *P. pastoris*, synthesized, and cloned into the pJ912 vector by DNA 2.0. The pJ912 vector is designed for the homologous recombination of the gene downstream of the methanol inducible AOX1 promoter. Furthermore, it adds an N-terminal *Saccharomyces cerevisiae* α-secretion signal to the recombinant protein. As such, gene sequences submitted to DNA2.0 were truncated to remove naturally occurring secretion signals as predicted by SignalP [29]. Codons necessary for a C-terminal 6His-tag were also included in the gene.

*P. pastoris* KM71H (Mut^s^ strain, i.e., slow growing on methanol) was transformed as per the manufacturer’s instructions for electroporation (Easy Select Pichia Expression Kit, Invitrogen, Life Technologies, Carlsbad, CA, USA) [40]. After electroporation, *P. pastoris* cells were first screened for resistance to the antibiotic zeocin as it selects for recombination of the pJ912 vector into the yeast’s genome. Resistant colonies were then transferred to agarose plates of buffered methanol-complex medium (BMMY) (1% yeast extract, 2% peptone, 100 mM potassium phosphate pH 6.0; 1.34% YNB; 4 × 10^−5^% biotin; 0.5% methanol, 10 g/L agar) in a grid pattern. This approach allowed us to screen for heterologous protein production by an overlay immunoassay. Two *P. pastoris* clones that had been transformed with the empty pJ912 vector were also spotted onto the plate in opposite locations to serve as negative controls. In this assay, the spotted regions in a grid formation were allowed to grow into patches over 2 days at 28 °C. Thereafter, a nitrocellulose membrane (Nitrocellulose transfer membrane 0.45 μm, BioRad, Hercules, CA, USA) was laid over the cell patches and incubated at the corresponding growth temperature for 2 h. The membrane was then rinsed with water to remove cellular debris, and a procedure similar to that for a traditional Western blot was applied targeting the His6-tag of the protein. First, the membrane was washed with TBST (20 mM Tris-HCl, 137 mM NaCl, 0.1% Tween 20, pH 7.5) for 5 min on a shaking platform at room temperature. Then, the membrane was incubated for 1 h in blocking buffer (5% skim milk) on the shaker at room temperature. The membrane was then very briefly rinsed with water to remove the blocking buffer, then immediately transferred to TBST containing the primary antibody against the C-terminus His6-tag (Mouse anti-his, Thermo Fisher Scientific), then incubated for 1 h at room temperature on a shaker. The membrane was then washed three times with fresh TBST to be then transferred to the secondary antibody in TBST (Goat anti-mouse anti-his alkaline phosphatase, Thermo Fisher Scientific, Waltham, MA, USA). The membrane was incubated in this solution for an hour at room temperature on the shaker. Then, the membrane was washed three times with TBST for 5 min each. Finally, the membrane was transferred to the BCIP/NIP solution (5-bromo-4-chloro-3-indolyl phosphate/nitro blue tetrazolium, SERVA electrophoresis, Heidelberg, Germany) (one tablet per 10 mL of water as per manufacturer’s instructions). Once the purple colour of the reaction developed (after approximately 20 min), it was stopped with water.

Twelve of the colonies that produced the purple colouring indicating secretion of the heterologous protein were further screened for production levels. As such, 50 mL cultures of these colonies were grown in BMGY (1% yeast extract, 2% peptone, 100 mM potassium phosphate, pH 6.0 1.34% YNB, 4 × 10^−5^% biotin, and 1% glycerol). After approximately 24 h, the BMGY culture medium was replaced with BMMY medium, which induces production of the heterologous protein. Over 4 days, a 1 mL sample was taken from each flask each day and screened for protein in two ways: the samples were spun down in a table-top centrifuge to remove cells, and the culture supernatant was analysed both by silver-stained SDS-PAGE gel (Laemmli system) and a Western dot plot.

The Western dot plot was conducted in the same way as the overlay assay, except the culture supernatant was pipetted in 1 μL increments onto the nitrocellulose membrane for a total of 20 μL per spot. The *P. pastoris* colony that produced the darkest purple was chosen to produce the recombinant protein for the study, as the results of the SDS-PAGE gel were inconclusive in each case.

### 2.3. Flask Protein Production

Protein production in Erlenmeyer flasks was conducted as described by the manufacturer (EasySelect Pichia Expression Kit, Invitrogen, Life Technologies, Carlsbad, CA, USA). Using a single clone, 25 mL BMGY was inoculated and grown overnight in a shaking incubator at 28 °C and 300 rpm. Once the culture reached an optical density (OD) between 2 and 4 at a wavelength of 600 nm, it was used to inoculate a 1 L culture of BMGY in a 4 L baffled flask whose opening was covered by multiple layers of cheesecloth. This larger culture was left to incubate at 28 °C and 300 rpm until it reached log phase growth (OD between 2 and 6). The cells were then harvested in sterile centrifuge bottles spun at 1500× *g* for 5 min at room temperature and resuspended in 500 mL BMMY to induce heterologous protein production. The culture was left to incubate under the same conditions for three days before the culture supernatant was harvested for purification of the secreted protein. Methanol (100%) was added to 0.5% of the culture’s volume every 24 h until the protein was harvested on day four.

### 2.4. Bioreactor Protein Production

All bioreactor protein production runs were performed using a Biostat B Plus bioreactor (Sartorius, Göttingen, Germany) equipped with pH, oxygen, and foam probes from Hamilton and connected to a MFCSwin process control system (BBI B. Braun Biotech International, Melsungen, Germany). The methods described in the Invitrogen Life Technologies Pichia Fermentation Process guidelines Version B053002 were applied with modifications as described in [41]. Briefly, for each run, 1 L of fermentation basal salts medium was prepared (26.70 mL 85% phosphoric acid, 0.93 g calcium sulphate, 18.20 g potassium sulphate, 14.90 g magnesium sulphate-7H_2_O, potassium hydroxide 4.13 g, 40.00 g glycerol); 100 mL was then put aside, and the remaining 900 mL placed in the bioreactor for autoclave sterilization. After sterilization and cooling, the pH of the reactor was aseptically adjusted to 5.0 with 28% ammonium hydroxide; the pH probe was calibrated as per the manufacturer’s instructions prior to sterilization.

Once the pH was adjusted, 4.35 mL of PTM_1_ salts were aseptically added to the bioreactor (6.0 g/L cupric sulfate-5H_2_O, 0.08 g/L sodium iodide, 3.0 g/L manganese sulfate-H_2_O, 0.2 g/L sodium molybdate-2H_2_O, 0.02 g/L boric Acid, 0.5 g/L cobalt chloride, 20.0 g/L zinc chloride, 65.0 g/L ferrous sulfate-7H_2_O, 0.2 g/L biotin, 5.0 mL sulfuric acid). The oxygen probe was then calibrated for 100% (maximum agitation and aeration with air) and 0% (maximum agitation and aeration with nitrogen).

To begin production, a 100 mL *P. pastoris* culture grown in BMGY was gently spun down (10 min, 24 °C, 4000 g) and resuspended in the remaining 100 mL basal fermentation salts that had been passed through a 0.22 μm pore size filter (Millex-GP). The cells were then added aseptically to the bioreactor. Set points of a pH of 5.0, 20% dissolved oxygen, and a temperature of 28 °C were used throughout the cultivation. The dissolved oxygen concentration was maintained above 40% by automatic cascade stirring and gas flow feedback control using the proportional–integral–derivative (PID) controller function in the bioreactor. Specifically, the stirrer speed was first increased from 300 to 1200 rpm and then the airflow was increased from 0.5 to 3.0 L/min until the maximum oxygen transfer capacity of the reactor was reached. Antifoam (Struktol J 647) was added automatically as required. The pH was maintained by addition of 15% ammonium hydroxide and 15% phosphoric acid.

Once the glycerol in the batch was fully consumed as indicated by a spike in dissolved oxygen with minimal aeration, the density of the culture was further increased by a glycerol feed (15 mL/h, 50% (*w*/*v*) glycerol with 1.2% (*v*/*v*) PTM_1_ trace salts). A glycerol-fed batch was conducted until the optical density in the reactor was approximately 200. Thereafter, the glycerol-fed batch was stopped and the methanol-fed batch begun to induce protein expression (AOX1 promoter) (3 mL/h, 100% methanol with 12 mL/L PTM_1_ salts). The flow rates of both feeds were adjusted so as to avoid an accumulation of either glycerol or methanol in the reactor. This was determined by stopping the feed and observing whether the metabolic rate of *P. pastoris* decreased or remained constant.

The cultivation was stopped and the protein harvested once oxygen consumption began to decrease (2–3 days after induction). The cultivation broth was spun down twice to remove the cells (12,000× *g*, 12 min, 4 °C) in preparation for nickel affinity purification.

### 2.5. Nickel Affinity Purification of Hydrophobins

In the flask production trials, batch nickel-affinity purifications were conducted as per the manufacturer’s instructions (QIAExpressionist, Qiagen, Hilden, Germany).

In the bioreactor production trials, the pH of the harvested culture supernatant was adjusted to a pH of 7.8 with 5 M NaOH on ice. Precipitated salt was then removed by centrifugation at 5 °C at 8000× *g* for 10 min. The supernatant was then pumped onto the His-Trap FF crude pre-packed 5 mL columns (GE Healthcare Life Sciences, Chicago, IL, USA) at a rate of 5 mL/min that had been equilibrated prior with five column volumes of the binding buffer (20 mM Na-phosphate, 500 mM NaCl, 20 mM imidazole pH 7.4). In contrast to many other pre-packed columns, the “crude” column does not require that the sample be filtered prior to its application. As such, potential filter–hydrophobin interactions were avoided. Once the sample was pumped onto the column, it was rinsed with 100 mL binding buffer. Then, the binding buffer was changed to the elution buffer (20 mM, 500 mM NaCl, 500 mM imidazole pH 7.4) and 5 mL fractions were collected for SDS-PAGE analysis. Purified protein samples were then stored in the fridge at 5 °C. The sample was not filtered prior to purification.

In the samples derived from the flask production trials, all buffer exchanges were conducted using the Spin-X UF concentrators (Corning, Corning, NY, USA) with a 5 kDa molecular weight cut-off PES (polyethersulfone) membrane. In the case of the bioreactor production trials, all buffer exchanges were conducted with the use of Econo-Pac 10DG columns (Bio Rad, Hercules, CA, USA) as per the manufacturer’s instructions.

### 2.6. SDS-PAGE

In the analyses of proteins produced in the 50 mL flask cultivations, SDS-PAGE was run using the standard Laemmli method as described in [42].

Nu-PAGE Bis-Tris Mini Gels (4–12% gradient) and NuPAGE LDS sample buffer with NuPAGE reducing agent (Life Technologies, Carlsbad, CA, USA) were used for the analysis of samples derived from the 500 mL cultures and bioreactor productions. Furthermore, the accompanying NuPAGE MES SDS running buffer (Life Technologies) was used.

### 2.7. Protein Concentration Determination

A total amino acid composition analysis (TAACA) was conducted at the Hospital for Sick Children at the SickKids Proteomics, Analytics, Robotics & Chemical Biology Centre (SPARC BioCentre) (Toronto, ON, Canada). Prior to submitting the sample for analysis, the buffer of the protein sample was exchanged with 10 mM ammonium acetate using a desalting column (Bio-Rad Econo-Pac 10DG Desalting column).

## 3. Results and Discussion

### 3.1. Selecting Hydrophobin Targets for Expression in P. pastoris

A survey of the literature reveals a total of six hydrophobins that have been solubly expressed by various strains of *P. pastoris*: the greatest number for a single heterologous host. These are summarized in Table 1. When yields are reported, they range from 80 to 300 mg/L culture medium, and are greatest when a bioreactor is used as opposed to a shake flask. It is important to note, however, that this table is not provided to compare different approaches to strain construction, but rather to gain a sense of the yields that can be achieved with this host. The effect of strain design cannot be isolated from the different conditions for culture and purification chosen.

**Figure 3:**
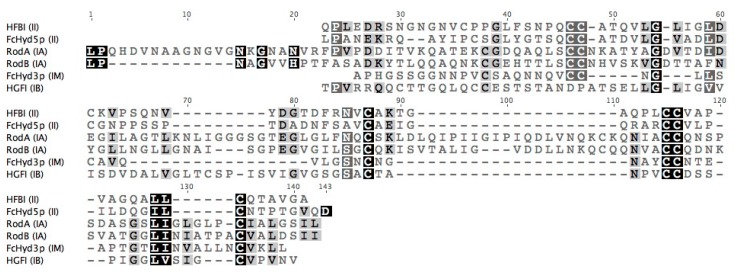
Amino-acid sequence alignment of Class I and II hydrophobins previously expressed by the host *P. pastoris* with the corresponding amino-acid sequence identity matrix in Table 2. Colours indicate global consensus: high agreement to lower agreement are represented from black to light grey respectively.

In these studies, soluble hydrophobins from across the spectrum of Class I and II protein sequences offered by fungal genomes were produced as described in the protein sequence alignment of Figure 3 and Table 2. While the Class II hydrophobins FcHyd5p and HFBI share 37% identity, the others belonging to Class I share no more than 18% identity. This is to be expected, given that Class I hydrophobins contain two highly distinct protein sequence-based subdivisions (IA and IB) [25]. Each is represented amongst the hydrophobins of Table 1 as can be seen when their positions are indicated on the SA-PCA of Figure 2 established in [25]. In this study, PCA was used as a means of visually representing the relationships between the different protein sequences in a large data set (1046 sequences from 215 fungal species). Briefly, the protein sequences that cluster together along a principal component tend to share similarities in their predicted primary sequences, the degree of similarity for a given group increasing with distance from the origin of the graph. The differences described in the second principal component (PC2) subdivide Class I hydrophobins into three groups: IA, IB, and IM. While these groupings were first observed in the PCA, they were confirmed by conducting sequence alignments of these regions as well as by overlaying PFam predictions for Class I and II as seen in Figure 2.

**Figure 4:**
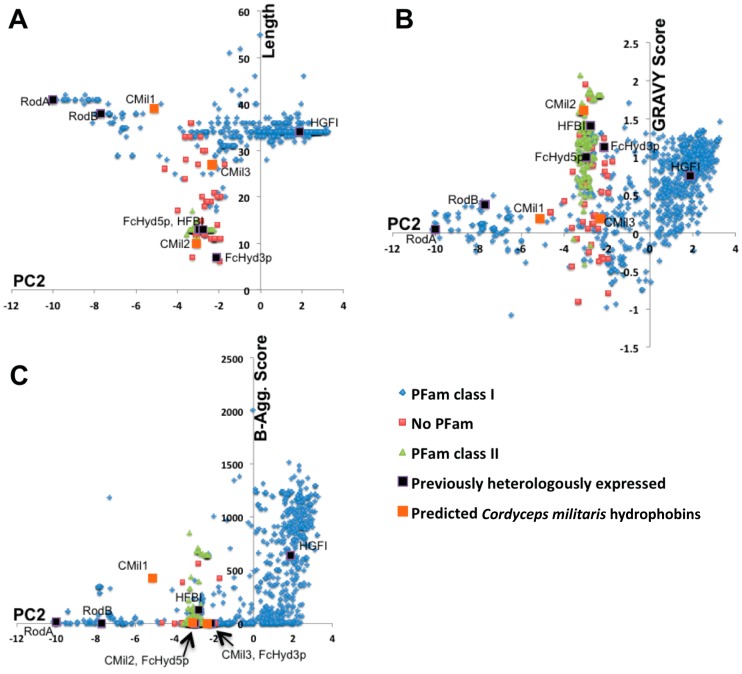
Length (**A**); Grand Average Hydropathy Score (GRAVY) (**B**); and B-aggregation scores (**C**) plotted against the values of PC2 in the SA-PCA illustrated in Figure 2. Lengths and scores are calculated over the region between the third and fourth cysteines of the conserved eight-cysteine pattern.

While it is well-accepted that the different physical properties of proteins are fundamentally reflected by differences in their primary sequences, the role specific amino acid residues play in the molecular function of hydrophobins remains unclear. The predicted properties of protein primary structures, such as length, grand average hydropathy (GRAVY) [33], and propensity to aggregate [34], are a function of position in the SA-PCA shown above. In Figure 4, these parameters are calculated only for the region between the third and fourth cysteines (C3-4 region) and are plotted against the values for PC2 (i.e., the principal component which subdivides Class I in Figure 2). The region between the third and fourth cysteines contributes to the protein sequence-based distinctions between Class IA and IB as established by the sequence alignments of the SA-PCA regions and reported in [25].

It is immediately clear for instance in Figure 4A that Class I subdivisions are based in part on the length of this region given that average lengths correlate to different regions of PC2. In Figure 4B, the GRAVY scores are plotted against PC2, negative scores representing hydrophilic regions and positive scores representing hydrophobic regions [33]. While Class IA sequences (negative PC2 values) are centered at 0, the theoretical hydrophobic/hydrophilic boundary, Class II and Class IB (positive PC2 values) sequences are on average far more hydrophobic over the C3-4 region. Finally, in Figure 4C, the propensity of this region to form β-sheets that aggregate is predicted with a score: the higher the score, the greater the propensity to form these structures. This score increases from the smallest to greatest PC2 values, predicting that this region may have a higher propensity to aggregate in Class IB sequences. While these predictions are highly speculative, they motivate further investigation to test whether specific solution properties can be correlated to the different regions in the SA-PCA.

To ultimately test this hypothesis, we began to establish a pipeline to heterologously express hydrophobins from across the SA-PCA. A total of four genes were chosen for strain construction: one confirmed hydrophobin gene and three predicted. The confirmed Class IA hydrophobin RodA (*Aspergillus fumigatus*) was chosen to serve as a positive control given its previous high-yield soluble expression in *P. pastoris* X33 (200–300 mg/L culture) [43]. The three predicted genes chosen are the complete set of hydrophobins contained in the *Cordyceps militaris* genome.

**Figure 5:**
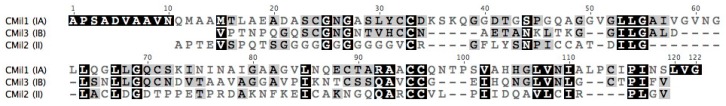
Sequence alignment of the hydrophobins predicted from the *C. militaris* genome. Colours indicate global consensus: high agreement to lower agreement are represented from black to light grey respectively.

*Cordyceps* (order Hypocreales, class Sordariomycetes, phylum Ascomycota) is a fascinating genus of filamentous fungi encompassing a large and diverse number of entomopathogenic species (i.e., parasites of insects and other arthropods) [44]. Each species is highly specific to the host it infects, in certain cases targeting a single sub-species of insect; however, the underlying mechanism regulating this specificity remains unknown. Given recent advances in understanding the role of the Class I hydrophobin MPG1 (*Magnaporthe grisea*) in surface interactions related to the infection of rice [12], it is conceivable that *C. militaris* hydrophobins could play similar roles in recruiting the catalytic enzymes required for infection to the surface of the insect. A first step, however, to testing this hypothesis is the soluble expression of these proteins, our aim in this article. Furthermore, in the context of our study, each of the *C. militaris* genes is predicted to encode for a hydrophobin belonging to a different Ascomycota Class I subdivision as well as Class II: CMil1 to Class IA, CMil2 to Class II, and CMil3 to the IM region (Table 3, Figure 5). These proteins have not been previously studied. Furthermore, this is the first study to express heterologously in the same host proteins belonging to each subdivision.

### 3.2. Approach to Recombinant Production of Selected Hydrophobins

**Figure 6:**
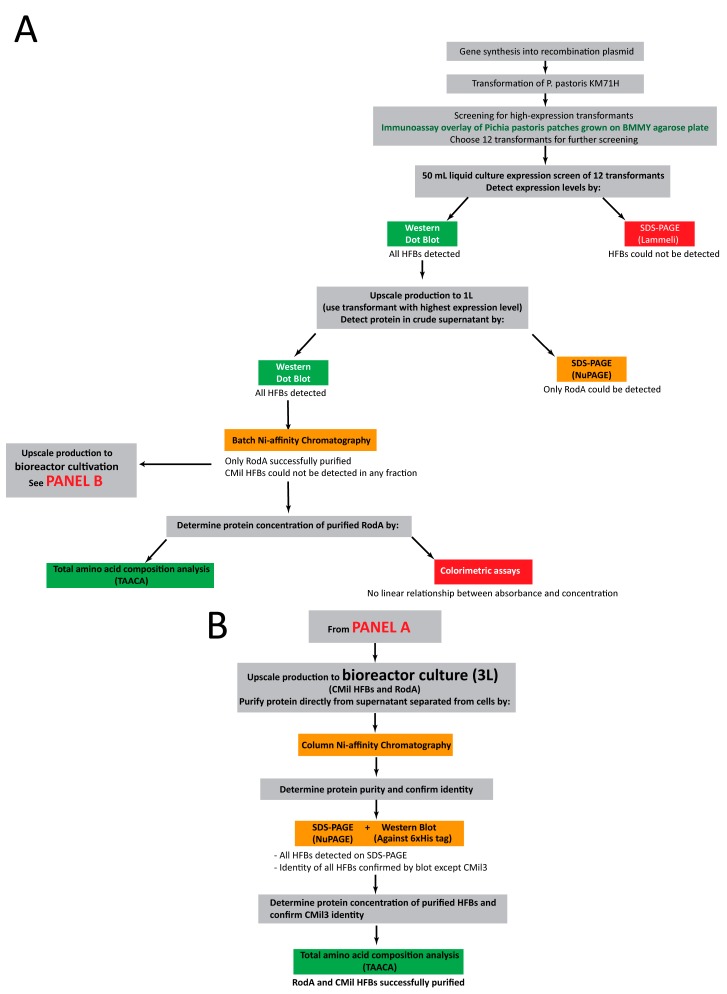
Flow chart describing the approach to the recombinant expression and purification of hydrophobins (HFBs) in *P. pastoris* KM71H for (**A**) expression in flasks and (**B**) bioreactor expression.

An overview of the approach taken to develop the hydrophobin production pipeline is described in the flow chart in Figure 6*.* While certain native hydrophobin secretion signals have been proven to be effective in *P. pastoris*, these were replaced by the *Saccharomyces cerevisiae* α-secretion signal given its proven consistent effectiveness in this host organism [45]. Secreted expression of the protein results in the accumulation of the recombinant protein in the bulk solution of the culture. In contrast to intracellular expression, the protein concentration remains relatively dilute, which may improve solubility. Furthermore, given that *P. pastoris* naturally secretes few endogenous proteins, secretion of the recombinant protein may facilitate downstream processing, such as purification. The secretion peptide is cleaved as the protein is transported to the exterior of the cell [40].

To facilitate detection and purification, a region encoding a C-terminal 6His-tag was included in the gene construct. In the hydrophobin structures that have been experimentally resolved, both the N-terminal and C-terminal regions are exposed to the bulk solution [12,25,46,47,48,49]. In the case of Class I proteins, however, the N-terminus may be involved in assembly [46]. As such, to minimize the likelihood of influencing protein assembly, the 6His tag was placed at the C-terminus. It is important to note that while in [35] the presence of a C-terminal 6His tag reduced the ability of FcHyd5p produced in *P. pastoris* to provoke “gushing” in beer, it did not alter any of its other properties, such as its ability to reverse the wettability of hydrophobic surfaces and stabilize the air bubbles in the culture medium.

Strain KM71H (Mut^s^, slow growing on methanol) of *P. pastoris* was chosen, as this strain allows for the methanol accumulation necessary for the activation of the promoter in shake flasks when it is only dosed daily. Furthermore, this restrains the culture densities to those that are manageable under the limited oxygen mass transfer of a shake flask. Industrially, this strain requires less methanol and less cooling during induction and protein production.

It is important to note that this study concentrates on the recombinant expression and detection of hydrophobins, and not functional characterization. This is partly because hydrophobin function is not strictly defined. It can range from the ability to form amyloid-like aggregates at the air–water interface to simply self-assembling on a surface. Evaluating whether a heterologously expressed hydrophobin is active or not is only possible if a molecular function is well-defined. As such, we limit the scope of this article to the soluble expression of hydrophobins for the discovery of molecular functions and potential applications.

### 3.3. Initial Screens of Hydrophobin Production are Enabled through Dot Blot Analyses

The pJ912 vector used to construct the strains is designed to recombine downstream of the AOX1 promoter in the *P. pastoris* genome, and as such, multiple recombination events can occur [40]. The expression levels of the recombinant gene therefore vary both with location and copy number in the genome. Thus, individual transformants must be screened to select the individual with the highest expression levels. Two methods were used to screen transformants for high expression in the crude 50 mL cultures: SDS-PAGE and dot-blot.

In the case of SDS-PAGE, the Laemmli method was used to separate the protein on the gel, then a silver stain was applied to detect protein. Silver stain is one of the most sensitive colorimetric assays used to detect protein (low ng range) [50]. While the exact mechanisms by which silver ions interact with protein remain unknown, it is seemingly independent of amino acid composition [51,52]. This is of significant importance given the relatively low binding hydrophobins to, for instance, coomassie. Such dyes are thought to bind to protein through van der Waals interactions as well positively charged amine groups (e.g., with lysine and arginine) [52], which are typically in low abundance in known hydrophobin sequences.

Hydrophobins could not be detected in the crude culture of the transformants by this method of SDS-PAGE gel. It is interesting to note that when both the stacking and resolving sections of the gel were stained, thick bands were detected in the stacking gel, suggesting the presence of protein assemblies whose size or charge did not allow migration into the resolving gel [53]. While these bands were found exclusively in lanes that were loaded with the crude supernatant and not those of the control culture, the multiple protein bands complicated estimates of relative expression levels for the tested transformants. These initial observations serve to underscore that the chemistry of hydrophobins poses a number of challenges for SDS-PAGE. Their assemblies are to varying extents SDS-insoluble, and the structures of their monomers are highly stable and characterized by a specific pattern of disulphide bridges [54]. As such, they are prone to refolding in the gel and forming disulphide bonds under the basic conditions of the Laemmli buffers [15,52]. While this can lead to restricted migration in the stacking gel, this can also lead to the presence of multiple bands for a single protein in the separating gel.

By contrast, the recombinant expression of the selected hydrophobins was confirmed by dot blot through an immunoassay of the 6His-tag. The reported sensitivity of the dot blot is generally of the same order as the silver stain previously described, but can detect as much as 0.1 ng under optimal conditions [55]. The crude protein solution is, however, placed directly on a membrane that is dried then assayed. As a result, protein solubility and aggregation are not concerns as they are in SDS-PAGE. For each hydrophobin, the transformant that produced the culture that developed the deepest colour was considered the most promising and was chosen for further investigation.

### 3.4. Protein Production in 500 mL Cultures in Flasks

The protein production of transformants chosen in 4.4 was upscaled to 500 mL cultures in 4 L baffled flasks to improve aeration of the culture. Greater dissolved oxygen leads to the maintenance of a culture with a higher cell density, which can lead to enhanced protein expression, potentially facilitating detection.

The crude culture supernatants were analyzed by SDS-PAGE, this time with a specialized gel system: NuPAGE, also known as Sauer bis-Tris [56]. In contrast to the previously used Laemmli system, the NuPAGE running conditions are slightly acidic, contributing to the maintenance of the reduced state of the disulphide bridges. LDS is used instead of SDS as this detergent potentially better solubilizes the hydrophobins at a slightly acidic pH. Furthermore, this detergent binds to protein in a higher stoichiometric ratio than SDS [56]. Gradient gels were used, as they enhance the resolution of separation.

**Figure 7:**
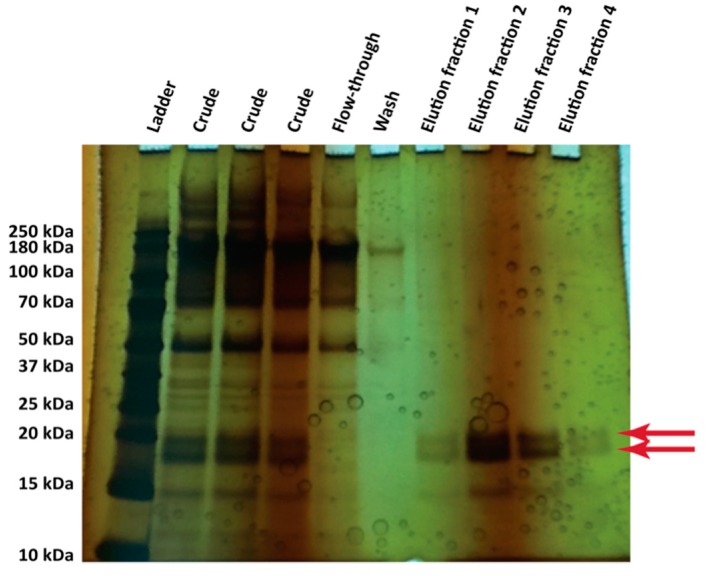
Silver-stained SDS-PAGE gel (20% gradient NuPAGE system) of the purification fractions from the culture supernatant of *P. pastoris* KM71H transformants expressing recombinant hydrophobin RodA (*A. fumigatus*). A pre-stained ladder was loaded (6 μL), and 23 μL of each sample was loaded into each lane. The predicted molecular weight of RodA is 15.2 kDa. The identity of these bands as RodA at these locations was confirmed by mass spectrometry in [38]. These are likely glycoforms of the protein [57].

With these alterations to the methods, a single hydrophobin (RodA) could be detected in the crude supernatants of the 500 mL flask cultures (Figure 7, “crude” lane). RodA was therefore purified from the culture using nickel affinity purification (Figure 7). Two bands were observed, both previously confirmed to be RodA in [38] and likely glycoforms of the protein [57].

These expression trials with RodA indicate that improvements to *Pichia pastoris* cultivation, in combination with changes to gel electrophoresis and detection of the produced protein, can facilitate the tracking and recovery of recombinantly expressed hydrophobins. It was hypothesized that for the *C. militaris* hydrophobins, either the yields were still not sufficiently high for their detection or the aggregation of the protein occurred under the solution conditions in the rich medium used. As such, the use of a bioreactor system was explored as a means of further improving protein yields and testing this hypothesis.

### 3.5. Bioreactor Production

Accordingly, the production of all four proteins was scaled up to a 3 L bioreactor to provide the aeration necessary for a high-cell-density cultivation (i.e., ≈400 g/L wet cell mass). A minimal salt media was used in contrast to the complex rich medium used in shake flasks, and the pH was reduced from 6.0 to 5.0. The use of bioreactors for protein expression can significantly enhance protein yields. The growth and maintenance of dense *P. pastoris* cultures requires significant aeration that can be achieved by air filtered from the atmosphere or enriched in oxygen that has been sparged into the culture medium and is accompanied by mixing by an impeller. The optimal pH and temperature can be closely monitored and maintained as well as the optimal carbon source and inducer concentrations.

**Figure 8:**
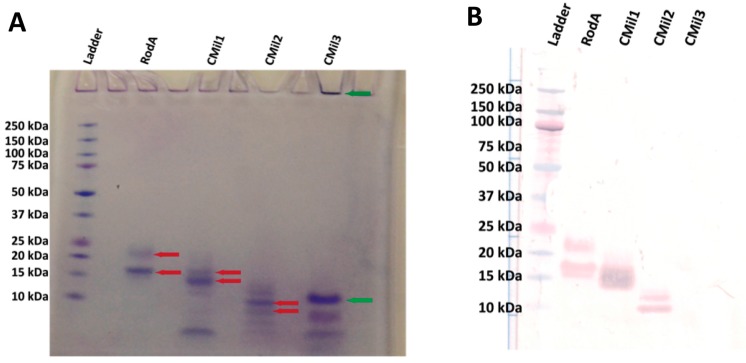
(**A**) Coomassie-stained (G-250) SDS-PAGE gel (20% gradient NuPAGE system) of the recombinant hydrophobins purified from bioreactor cultivation and (**B**) Western blot of SDS-PAGE gel against hydrophobin 6His-tag. Transferred gel was run under the same conditions as those for (**A**). A pre-stained ladder was loaded (3 μL) along with 23 μL of each sample. Red arrows in (**A**) indicate bands that are detected by the Western blot in (**B**). The green arrows indicate locations where CMil3 is believed to reside on the gel despite testing negatively in the Western Blot of (**B**). The green arrow at the top of the well indicates that there may be aggregation of the protein prior to or during the running of the sample on the gel. The predicted molecular weights of the proteins are as follows: RodA 15.2 kDa, CMil1 12.8 kDa, CMil2 10.0 kDa, and CMil3 9.7 kDa. Glycosylation likely accounts for the higher-than-expected position of RodA on the gel (between 15 and 20 kDa) [38,57].

At the time of purification, the pH of the culture supernatant was adjusted then pumped directly on a nickel affinity chromatography column without any additional clarification other than centrifugation. This approach allowed us to avoid any buffer exchange or concentration step that could lead to increased protein aggregation or adhesion to membranes. Together, these methods resulted in all four hydrophobins visible on an SDS-PAGE after purification at mg/L culture yields (Figure 8A, Table 4). Yields were calculated based on total amino acid composition of the samples and reported as mass of purified protein per volume crude supernatant processed from the bioreactor. Whereas the yields obtained as described in Table 4 are at least an order of magnitude lower than those achieved in the bioreactor cultivations in the literature (Table 1), it is important to recognize that the yields achieved herein are prior to optimization.

RodA was again purified to its two bands as described in the literature and confirmed by Western blot (Figure 8A,B). The three predicted *C. militaris* hydrophobins from *C. militaris* (CMil1, CMil2, and CMil3) were also solubly expressed and purified. While they could not be detected by SDS-PAGE gel in the crude supernatant [58], they were readily detected once purified using the same NuPAGE system. Major bands corresponding to the approximate predicted molecular weights of CMil1 and CMil2 are present on the gel (Figure 8A), and there are other bands that could correspond to degradation products or contaminating proteins. In the case of CMil3, three major bands range from above to below its predicted molecular weight of 10 kDa (Figure 8A). While the major bands in the CMil1 and CMil2 lanes were confirmed by Western blot, none of the bands present in the CMil3 lane reacted to this immunoassay despite detection by dot blot (Figure 8B).

**Figure 9:**
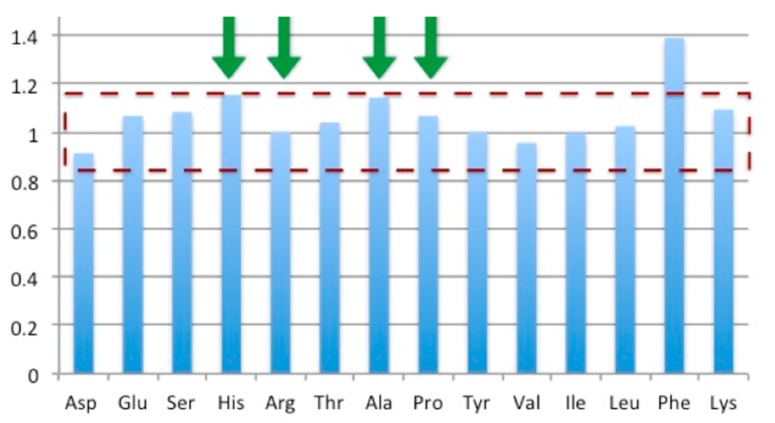
The amino acid composition analysis of CMil3 reveals the ratio of predicted and measured amino acids in the sample as presented on the y axis. The average of the ratios for the most stable residues during hydrolysis (histidine (His), arginine (Arg), alanine (Ala), and phenylalanine (Phe)) is 1.07 with a standard deviation of 0.11. This suggests that the major bands on the gel can be attributed to Cmil3.

A total amino acid composition analysis allows for both the concentration determination of a sample as well as the evaluation of its purity. Essentially, in this analytical technique the protein is acid-hydrolyzed and the mass of individual amino acids is quantified. While the total mass of amino acids corresponds to the total mass of protein in a given sample, the comparison of the experimental ratio of the individual amino acids to the expected ratio provides a measure of its purity. The purity of the CMil3 solution was therefore verified by this technique (Figure 9). The ratio of the expected amino acid content over the detected content was 1.07 with a standard deviation of 0.11. As such, these bands can likely be attributed to CMil3. It is possible that the chemical nature of CMil3 does not allow for a proper transfer to the Western blot membrane, resulting in the false negative.

## 4. Conclusions

In this study, hydrophobins from across the spectrum of sequences offered by fungi of the Ascomycota phyla were solubly expressed using a method that eliminated steps such as the dissolution of inclusion bodies in trifluoroacetic acid. Furthermore, secretion of the protein in the culture medium likely contributed to the ability to produce soluble proteins without the use of a solubilisation tag. More importantly, however, this work has not only made possible the characterization of the solution and interfacial properties of the entire set of predicted *C. militaris* hydrophobins, but has also established a framework to tackle the pipeline production of hydrophobins. Given the wide range of their solution behaviours, it is likely that the heterologous production of novel hydrophobins will remain an iterative process. As their properties become known, their production and purification can be optimized. With validated strains in hand, future work should address the contribution of culture medium (i.e., minimal media versus rich media) to the ability to purify the CMil proteins as well as other changes in culture conditions applied in this study.

The inclusion of a tag, such as the 6His-tag in the constructs of this study, is essential to not only screening transformants for expression levels at a small scale, but also in tracking the protein while developing the methodologies for optimal expression at a large scale and subsequent purification. It is our experience that the detection of hydrophobin expression can also be promoted by the use of gel systems, such as NuPAGE, that seem to facilitate hydrophobin solubility and migration through the gel during electrophoresis. Consistent with the literature, cultivation in bioreactors increased recombinant protein yields. Together, these results demonstrate the need for the straightforward detection and quantification of hydrophobins. For example, without the ability to effectively detect these proteins at low scales, it is easy to assume that a given expression system is not an effective producer even though this may not be the case. While the inclusion of a tag allows for a qualitative assessment of protein levels, currently, a total amino acid composition analysis is the only method that allows for the accurate quantification of hydrophobins. Regardless, this study strengthens the claim in the literature that *P. pastoris* is a suitable organism for the production of hydrophobins across the spectrum of sequences displayed by the SA-PCA for the discovery of new molecular functions and application in biotechnology.

## Figures and Tables

**Table 1:** Class I and II hydrophobins previously expressed using the host organism *Pichia pastoris* secreted into the culture medium as described in the literature [35,36,37,38,39].

Protein	Class	Organism/Phylum	Yield *	Strain	Promoter	Tags	Culture Type ****	Notes on Protein Detection	Ref.
FcHyd5p	II	*Fusarium culmorum*/ Ascomycota	Not reported	X33	AOX1 **	No tag C-terminal 6His	Flask	Protein could not be readily detected without immunoassay against 6His tag	[35]
FcHyd3p	IM	*Fusarium culmorum*/ Ascomycota	Not reported	X33	AOX1	No tag	Flask	Transformants were screened by RT-PCR for high expression as protein could not be readily detected on gel	[36]
HFBI	II	*Trichoderma reesei/* Ascomycota	Not reported 120 mg/L	SMD1168H GS115	GAP *** AOX1	No tag No tag	Bioreactor Flask	Proteins detected by silver-staining of SDS-PAGE gels	[37]
RodA	IA	*Aspergillus fumigatus*/ Ascomycota	329 mg/L	X33	AOX1	C-terminal 6His	Bioreactor		[38]
RodB	IA	*Aspergillus fumigatus*/ Ascomycota	262 mg/L	X33	AOX1	C-terminal 6His	Bioreactor		[38]
HGFI	IB	*Grifola frondosa*/ Basidiomycota	86 mg/L	GS115	AOX1	No tag	Flask		[39]

* Yields are calculated as the total mass of the heterologous protein divided by the volume of the culture medium, ** Alcohol oxidase 1 promoter, *** Glyceraldehyde-3-phosphate promoter, **** When a culture type was not reported it was assumed to be in shake flasks.

**Table 2:** Amino-acid identity matrix of Class I and II hydrophobins previously expressed by host *P. pastoris*.

	HFBI (II)	FcHyd5p (II)	RodA (IA)	RodB (IA)	FcHyd3p (IM)	HGFI (IB)
HFBI (II)	-	37	14	12	17	17
FcHyd5p (II)	37	-	13	12	13	11
RodA (IA)	14	13	-	39	14	13
RodB (IA)	12	12	39	-	18	17
FcHyd3p (IM)	17	13	14	18	-	16
HGFI (IB)	17	11	13	17	16	-

**Table 3:** Amino acid identity matrix of the hydrophobins predicted from the *Cordyceps militaris* genome.

	CMil1 (IA)	CMil3 (IB)	CMil2 (II)
CMil1 (IA)	-	30	14
CMil3 (IM)	30	-	24
CMil2 (II)	14	24	-

**Table 4:** Yield of hydrophobins purified from bioreactor cultivations.

Protein	Yield *
RodA	4.6 mg/L
CMil1	3.6 mg/L
CMil2	4.1 mg/L
CMil3	8.3 mg/L

* Yield was determined by dividing the total mass of amino acids as determined by the total amino acid composition analysis by the volume of crude medium at the end of the cultivation.
